# Beyond swimming: emerging parameters for predicting the fertility of mouse spermatozoa

**DOI:** 10.1038/s41684-025-01647-9

**Published:** 2025-11-26

**Authors:** Nataliia Shapovalova, Thorsten Buch, Heinrich Bollwein, Johannes vom Berg, Eleni Malama

**Affiliations:** 1 Institute of Laboratory Animal Sciences, University of Zurich, Zurich, Switzerland; 2 Clinic of Reproductive Medicine, Vetsuisse Faculty, University of Zurich, Zurich, Switzerland; 3 AgroVet-Strickhof, Vetsuisse Faculty, University of Zurich, Lindau, Switzerland; 4 PhD Program in Biomedicine (BioMed), University of Zurich, Zurich, Switzerland

**Keywords:** Predictive markers, Assay systems, Flow cytometry, Urogenital models, Animal biotechnology

## Abstract

Cryopreservation of spermatozoa can be used as a cost-effective way of preserving the ever-increasing number of genetically modified mouse lines. Nevertheless, discontinuing the breeding of a line or strain is only warranted after the quality control of cryopreserved sperm; the ability of sperm to survive the freezing–thawing process and produce well-developed embryos needs to be confirmed. In animal research husbandries, the fertility of frozen-thawed sperm is routinely tested using in vitro fertilization. However, this procedure requires euthanizing a considerable number of females to acquire a sufficient number of oocytes, contradicting the ‘reduction’ principle of the 3Rs (replacement, reduction and refinement). Therefore, the research community has an interest in replacing in vitro fertilization tests with proxies that can collectively characterize the fertilizing potential of mouse sperm. Methods such as computer-assisted sperm analysis and flow cytometry enable a precise and multiparametric approach to evaluate sperm quality, encompassing ‘traditional’ traits and the functional status of subcellular structures of sperm. Moreover, single-cell data can be processed with machine learning algorithms, offering a deeper insight into sperm physiology and functional heterogeneity. Despite the advancements made, many of these assays are still far from being used in mouse sperm quality control owing to their limited time- and cost-efficiency, the insufficiency of fertility validation studies, and the complex data analysis needed to identify fertility markers. The genetic and phenotypic diversity of different mouse strains and lines makes the establishment of a robust methodology for fertility prognostics even more challenging. Thus, this Review summarizes the available methods for assessing sperm functional characteristics in laboratory mice and discusses their contribution to fertility prognosis.

## Abstract (web summary)

Cryopreservation of spermatozoa preserves genetically modified mouse lines, but quality control is critical. Given that in vitro fertilization testing presents limitations, this Review explores other available methods to evaluate sperm quality.

## Main

Genetically engineered mouse (GEM) lines have been extensively generated for use in biomedical research. However, following the completion of the project for which they were designed, many GEM lines are not actively used in other ongoing experiments. Continuous animal breeding to maintain these lines requires considerable financial resources and human effort. Furthermore, it poses an ethical problem as these unused mice are eventually euthanized without direct use in research^1,2^, and the overproduction and underutilization of animals are clearly discouraged in the 3R (replacement, reduction and refinement) guidelines^2^. To address this issue, researchers have turned to gamete and embryo cryopreservation, allowing the discontinuation of maintenance breeding^3^. In addition, good research practices dictate the cryopreservation of genetic material of GEM for security reasons, such as protection from disease outbreaks, breeding failures or facility closures and to ‘leap back in time’, hence limiting genetic drift. The success of this approach depends on the ability to revive the mouse lines after thawing the cryopreserved material. Sperm is a heterogeneous cell system consisting of subpopulations of spermatozoa that vary in their ability to fertilize an oocyte^4,5^. The main challenge posed by cryopreservation is preserving all the functional characteristics of sperm necessary for successful fertilization. Despite extensive research on cryopreservation and its effects on the viability and functional status of spermatozoa, we are still far from standardizing the methodology that would allow the prediction of sperm survival rates post-thaw in mice^6^.

In addition to factors associated with the male animal, such as age, strain or health status, other factors related to the collection and processing of mouse spermatozoa can affect their function and fertility. These include the site and method of sperm collection, the composition of the sperm extender, the temperature and the incubation time until the sperm is frozen or analyzed. Indeed, deviations from the incubation temperature of 37 °C, a high sperm:extender dilution ratio and an extended incubation period before cryopreservation can impair motility^7^. As shown in rodents, sperm samples obtained after electro- or drug-induced ejaculation are of lesser quality^8,9^, whereas those collected from the caput epididymis contain a higher number of immature cells^10–12^ compared with the cauda epididymis and vas deferens. Therefore, the latter are usually preferred for sampling in postmortem mice^13^.

Traditionally, the evaluation of sperm quality has been based on the microscopic assessment of the morphological and motion characteristics of a sperm population. Notably, functional features other than sperm motility and morphology, such as plasma membrane integrity, can also be relevant to the performance of mouse sperm after in vitro fertilization (IVF)^14^. Nowadays, several methods already established in animal spermatology, such as computer-assisted sperm analysis (CASA) and flow cytometry, allow the simultaneous evaluation of multiple aspects of sperm function, including sperm viability, kinematic parameters, mitochondrial function and nuclear chromatin stability^15,16^. Flow cytometry provides objective and standardized measurements, reducing the subjectivity associated with microscopic sperm assessment. Furthermore, it enables high-throughput analysis, allowing the time-efficient examination of thousands of cells in a sperm sample^17^. In addition to the evaluation of sperm functional status in the form of total cell population metrics, CASA and flow cytometry further offer a deeper insight into the functional heterogeneity and dynamics of different sperm subpopulations^5,16,18,19^.

**Fig. 1:**
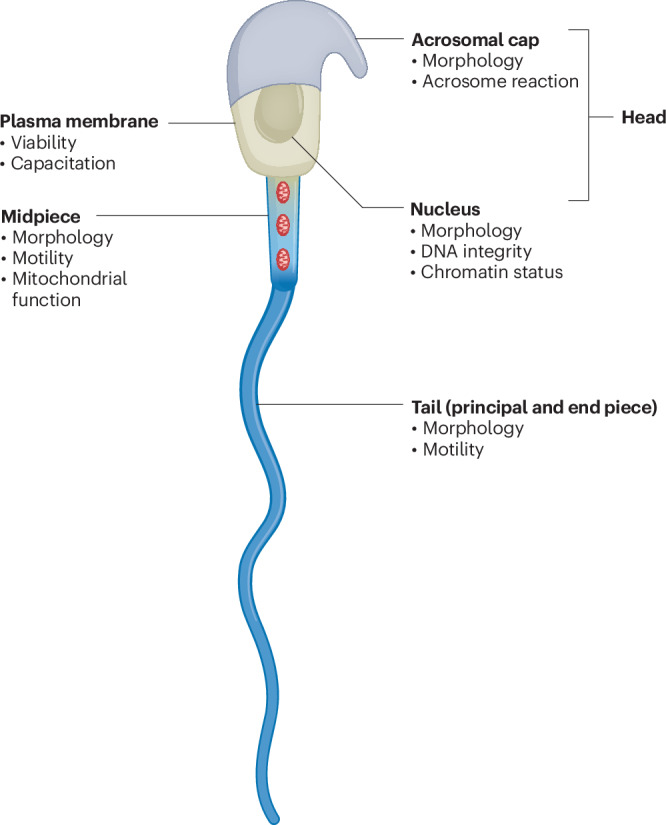
Aspects of sperm function related to male mouse fertility along with the major subcellular compartments associated with sperm functional characteristics. An illustration of a mature mouse spermatozoon, highlighting key functional traits relevant to fertilizing competence and mapped to their corresponding subcellular organelles or compartments: acrosomal cap, assessed for morphological integrity and its ability to undergo the acrosome reaction, which is essential for the penetration of the oocyte’s zona pellucida; plasma membrane, assessed for structural integrity (expressed as sperm viability) and membrane fluidity, which reflects the capacitation status of sperm; nucleus, linked to sperm head morphology and assessed for the condensation status of nuclear chromatin and the integrity of DNA; tail (midpiece), assessed for its contribution to the cell’s motility and energy production by mitochondrial function; tail (principal and end piece), assessed for morphological features influencing sperm kinetics and its contribution to the cell’s directional movement.

As described for other domestic animal species, a combination of sperm functional features, such as sperm motility, viability, acrosomal status, mitochondrial function and DNA integrity, can be used to develop an algorithm for male fertility prediction^16,20–22^. In recent years, researchers have also investigated several molecular markers to improve the predictability of sperm fertility^23–25^. Despite the substantial advancements in sperm functional analysis, we are still far from a standardized sperm quality control scheme, namely a validated set of quality criteria, that could reliably characterize the ability of mouse sperm to survive cryopreservation, produce a well-developing embryo and result in viable offspring. The small sample volume, the differences in the sperm processing protocols between facilities and the widely diverse genetic background of mouse strains represent additional challenges in mouse fertility prognostics. In this Review, we aimed to describe the functional characteristics of mouse spermatozoa that were shown to be relevant to their fertilizing potential (Fig. 1), as well as the methods (Table 1) by which these characteristics can be assessed for sperm quality control.

**Table 1:** Summary of the fertility-relevant sperm functional characteristics and the methods commonly performed for their assessment

Parameter	CASA	Brightfield microscopy	Fluorescence microscopy	Flow cytometry
Concentration	✓	✓	✓	✓
Morphology	✓	✓	✓	✓
Motility	✓	✓		
Hyperactivated motility	✓	✓		
Plasma membrane integrity		✓	✓	✓
Acrosomal status		✓	✓	✓
Mitochondrial membrane potential			✓	✓
Intracellular Ca^2+^ levels			✓	✓
DNA integrity		✓	✓	✓

## Sperm concentration

Measuring sperm concentration is one of the first steps in routine sperm analysis^26–28^. Given the small volumes usually obtained in mice, this quantitative characteristic is traditionally recorded as the number of cells per milliliter, per epididymis or the total number of cells per individual^28^. Sperm concentration reflects the efficiency of spermatogenesis^29^ and is critical for deciding whether a particular sperm sample can be used for further analysis and cryopreservation. For instance, in the case of oligospermic samples, low sperm concentration indicates against conventional cryopreservation, and the animal facility personnel should consider other alternatives to preserve genetic material, such as the cryopreservation of embryos produced with fresh sperm or even the less accessible options of freezing embryos produced by intracytoplasmic sperm injection (ICSI) and the single-cell freezing of sperm^30–33^. At this point, it is worth mentioning that ICSI and single-cell sperm freezing are technically challenging procedures that are not well established in most animal facilities.

Various techniques can be employed to estimate sperm concentration, including counting chambers, such as Neubauer hemacytometers^34^ and Makler chambers^35^, Coulter counters^7,34^, spectrophotometers^36^, flow cytometry^17,37^ and CASA^38^. The measurement of sperm concentration with an improved Neubauer hemacytometer remains the standard procedure for the calibration of other equipment used for the determination of sperm counts. In contrast to other methods, manual counting with cell counting chambers (with an etched counting grid), such as Neubauer hemacytometers, Makler, Thoma or Bürker chambers, is relatively inexpensive and requires only basic laboratory equipment, such as a phase-contrast microscope, and basic training; however, discrepancies between different types of counting chambers have been reported^35,39^. Concerns have been also expressed regarding the disagreement between sperm concentration measurements with counting chambers and disposable capillary-loaded slides of narrower depth typically used in CASA systems^34^. Kuster summarized that the sperm concentration of a sample is found lower when measured with CASA slides compared with counting chambers because of the Segre–Silberberg effect that occurs during laminar Poiseuille flow in capillary-loaded slides^34^. This results in an unequal distribution of particles and cells on a CASA slide, underestimating total cell counts. The assessment of concentration with counting chambers or CASA slides can be also affected by factors such as the dilution of sperm and technician-induced biases^36^. Sampling errors can be reduced by analyzing not less than 200 sperm cells per replicate (400 cells per sample) when using either a cell counting chamber or a capillary-loaded slide^27,36^. Notably, sperm concentration measurements with flow cytometry and automatic cell counters using fluorescence-based image cytometry, such as the NucleoCounter (ChemoMetec A/S, Allerød, Denmark), show higher repeatability compared with manual sperm counting and CASA^40^.

Different methods require different treatment of the sperm samples before assessment. A critical step when using a counting chamber is the immobilization of cells with fixing solutions to obtain accurate results. Automatic cell counters, for example, NucleoCounter instruments, require the use of a plasma membrane-disrupting reagent, which allows a DNA-binding dye, such as propidium iodide, to penetrate the cells and stain their nucleus before evaluation^36^. In contrast, there is no need for cell fixation when assessing sperm concentration with CASA. Nevertheless, DNA-binding dyes, such as Hoechst 33342, can help distinguish sperm cells from debris or extender particles, thereby considerably improving the accuracy of concentration measurements with CASA^41,42^. The ease of use and the capacity to simultaneously evaluate sperm kinematics render CASA an increasingly preferred platform for sperm concentration measurements^43,44^.

## Sperm morphology

Spermatozoa have a distinctive morphology that is well suited to their function. Mouse spermatozoa are characterized by a compact, hooked head, with an approximate head length of 7.5 ± 0.5 μm, and a long, thin tail (118.5 ± 1.6 μm)^45^. Sperm morphology and morphometrics are usually evaluated through microscopic observation^43,46^ or automated sperm morphometry analysis (ASMA) on a CASA platform^47^. Digital analysis of the sperm images captured with either method facilitates a more detailed assessment of sperm biometrics and the better identification of morphological abnormalities^48,49^. Offering detailed sperm biometric measurements, ASMA has promising potential for research fields, such as reproductive toxicology^50^ and evolutionary biology^51^. It also shows high repeatability and the potential to be coupled with differential stains (for example, SpermBlue)^52^ or fluorescent dyes for the simultaneous assessment of plasma membrane and acrosomal integrity (for example, propidium iodide and *Pisum sativum* agglutinin, respectively)^47^. Other methods, such as digital holographic microscopy^53^ and three-dimensional laser scanning microscopy^45^, provide a deeper insight into sperm ultrastructure and a better reflection of sperm fertilizing potential. Nevertheless, these imaging methods are still far from being part of the routine of assisted reproduction laboratories. Interestingly, as described in the work of Bulkeley et al. on equine sperm, imaging flow cytometry can also be applied to evaluate sperm morphology^54^. In this direction, Hernandez-Herrera et al. have recently advanced the deep-learning algorithm for the identification and characterization of sperm head and flagella in the mouse^55^. Thus, we believe that imaging flow cytometry shows promising potential for combining the assessment of sperm morphology and functional status. However, the equipment and expertise required for this method are available only in a few well-equipped laboratories, which limits its use mainly to research purposes and not to routine sperm quality control.

Spermatozoa with severe morphological abnormalities, either of the head or the tail, can display altered motion characteristics that compromise their ability to advance through the female genital tract, approach the oocyte and achieve fertilization^56,57^. In addition, some of these cells can have defective acrosomes, making it impossible to penetrate the oocyte membrane and fuse with it^58^. Therefore, sperm morphology is recognized as a key male fertility indicator. However, morphological traits cannot be predictive of male fertility per se, except for cases with a high percentage of abnormal cells in a sperm sample^59^. For sperm populations with only slight deviations from normal morphology and morphometry, further functional assays should be performed to more accurately assess their fertilizing ability.

In most species, high numbers of abnormal sperm are used to identify samples unsuitable for further processing and application in assisted reproductive technology (ART). In mice, the percentage of sperm with abnormal morphology can also be used to validate experimentally induced spermatogenic disorders and thus a successful experimental setup. Importantly, certain mouse strains and substrains, for example, C57BL/Kw, C57BL/6J, PL/J2-azh/azh, KE and BALB/c, can have high numbers of structurally abnormal spermatozoa^60–63^. For instance, C57BL/6J germ cells have a limited capacity to reduce their cytoplasm during spermiation, therefore showing a higher frequency of sperm morphological abnormalities compared with ICR mice^60^. Remarkably, sperm with severely aberrant morphology are still capable of producing viable embryos through ICSI. Burruel et al. reported that up to 91% of embryos produced by ICSI with severely abnormal BALB/c sperm (with 74% average incidence of misshapen heads) reached the blastocyst stage^64^. Nevertheless, approximately one-third (36–38%) of sperm with aberrant head morphology can have abnormal karyotypes that are evident at the metaphase of first cleavage in mouse embryos^65^.

## Sperm motility

The percentage of total or progressively motile cells in a sperm population is one of the most used indicators of the fertilizing potential of sperm or their ability to survive cryopreservation stress^66,67^. Although observation under a light microscope can be a fast and straightforward way to evaluate sperm motility, this can be prone to errors owing to its limited repeatability^68,69^. Tayama et al. reported the use of a sperm quality analyzer—a device that detects variations in the optical density caused by moving cells—to quantify the motility index in mouse sperm^7^. Nonetheless, detailed information about sperm kinematics, such as velocity values, wobble features and movement patterns, cannot be obtained through subjective motility evaluation with light microscopy or the sperm quality analyzer. This is possible using CASA, which enables an automated, rapid and objective analysis of sperm motion characteristics in a variety of species^15,70,71^. Owing to these advantages, CASA has become the most commonly used sperm analysis platform in assisted reproduction clinics and laboratories^28,72^. CASA platforms rely on specialized software to analyze digital images of sperm cells captured by a microscope equipped with a camera and provide a time-lapse view of sperm movement^73^. Recent advances in the CASA software algorithms for segmentation, localization and tracking of motile cells offer the opportunity to simulate different motility patterns^74^. Since sperm motion patterns can inherently vary between males of different GEM lines, CASA simulation models can make an important contribution to the comparative study of sperm motility between lines.

Sperm motion characteristics assessed with CASA have been linked to in vivo male fertility^75–77^ and the resistance of sperm to cryopreservation stress in the mouse^78^. Aberrant motility patterns and decreased sperm velocity are among the first manifestations of cell cryoinjury in frozen-thawed mouse sperm^78,79^. Evidence indicates that sperm of different mouse strains suffer motility deterioration after freezing to a varying degree^79–82^. One could argue that sperm motility deterioration induced by cryopreservation could be easily treated by sperm motility selection, a practice commonly applied before IVF to select and use only the motile fraction of a sperm population for the fertilization of the oocyte. Nevertheless, the efficiency of motility selection techniques can be strain dependent^80^. As reported by Szczygiel et al., motility selection can considerably improve the fertility of fresh and frozen sperm collected from C57BL/6J and BALB/c males; however, such an improvement is less evident in sperm produced from FVB and DBA/2 mice^80^.

Despite the advantages of CASA over subjective evaluation of sperm motility with brightfield microscopy, further improvement of CASA systems is necessary to assure the reliability and repeatability of the delivered data^72^. Factors such as the hardware and software settings^73^, the temperature of the media and equipment^83^, the composition of sperm extender (capacitating or noncapacitating)^71^, the chamber characteristics^84^ and the competence of the CASA operator can largely affect the output of the analysis^73,84^. Detailed information on the quality assurance of CASA systems has been published elsewhere^71^. Interlaboratory cross-validation is meaningful and should be performed in multicenter studies. Taken together, CASA facilitates the simultaneous measurement of sperm concentration, motility and biometric features (through ASMA), all of which are substantial components of semen analysis and reflect, at least in part, the functional status and fertilizing potential of a sperm population.

## DNA integrity

Along with a decline in motility and viability, cryopreserved mouse sperm can also show increased DNA fragmentation levels^66,81^. Therefore, changes in sperm DNA structure during freezing thawing are considered relevant to their ability to survive cryopreservation stress^85^. Chromatin integrity has been linked to other sperm functional features such as total and progressive motility, mitochondrial function, head morphology and sperm concentration^81,86,87^. However, deficiencies in DNA integrity can be present in otherwise normal and functional sperm^88^. Sperm with damaged DNA can successfully fertilize an oocyte, but the developmental potential of the early embryo can be compromised^89–91^. Previous studies have established a clear correlation between sperm DNA fragmentation and both genetic and epigenetic irregularities in mouse embryos^89,92^. The use of sperm with defective chromatin structure for IVF or ICSI—the method of choice for strains with profound male subfertility—can result in well-developing embryos, especially in cases where the DNA-repair mechanisms of the latter successfully minimize the damage sustained to embryonic DNA^93^. Nevertheless, it is likely that mouse embryos produced with DNA-fragmented sperm will show an increased risk for long-term pathologies of genetic origin in their postnatal life^89,92^. Therefore, evaluating sperm DNA fragmentation level is considered of major importance, especially in sperm samples used for ICSI.

Currently, various tests are available to evaluate sperm DNA damage, including direct (single-cell gel electrophoresis (Comet assay)^94^ and terminal deoxynucleotidyl transferase-mediated deoxyuridine triphosphate-nick end labeling (TUNEL) assay^95^) or indirect assays (acridine orange test, Sperm Chromatin Structure Assay (SCSA)^96^ and sperm chromatin dispersion test (SCD test)^97^). Direct methods involve the binding of a fluorescent probe to DNA strand break sites. In contrast, indirect methods detect the chromatin’s ability to disperse following experimentally induced protamine decondensation^98^. While the Comet assay is a fluorescence microscopy-based test, both fluorescence microscopy and flow cytometry can be used to perform the TUNEL assay^99,100^; the SCSA is exclusively a flow cytometric method.

The SCSA detects the presence of acid-induced DNA denaturation using the metachromatic properties and high chromatin specificity of acridine orange^101^. The standardized protocol and the repeatability of the assay, owing to its potential to assess high numbers of cells through flow cytometry, are some of the SCSA’s greatest advantages^102^. Thus, the SCSA is commonly the method of choice for routine monitoring of sperm DNA integrity in several animal species, including the mouse^103^. The SCSA requires a flow cytometer equipped with a blue laser, which is necessary for the excitation of acridine orange. Such equipment is nowadays becoming increasingly available in sperm laboratories or research units collaborating with animal facilities.

The TUNEL assay relies on the ability of a DNA-cleaving enzyme called terminal deoxynucleotidyl transferase to incorporate labeled nucleotides into the 3′ ends of fragmented DNA strands^95^. One can use a flow cytometer instead of a fluorescence microscope to increase the sensitivity and specificity of the TUNEL assay^99^. Nonetheless, compared with the SCSA, TUNEL is less efficient in staining sperm DNA breaks in compacted chromatin^103^. The SCD test is a user-friendly and cost-effective alternative to SCSA that only requires a light microscope^97^; however, it exhibits lower reproducibility than SCSA^104^. The SCD test is based on the loss of sperm chromatin structure after acid denaturation so that the DNA ring adheres to the residual nuclear structure to form the characteristic halo that can be observed with brightfield microscopy^97^. The extent of DNA fragmentation can also be quantified with toluidine blue staining^105,106^. Image cytometry algorithms can be applied to discriminate cell subpopulations with different spectral characteristics and thus diverse levels of DNA damage in toluidine blue-stained sperm^105^.

The Comet assay is widely used for somatic cells but can also be applied to spermatogonial and mature sperm cells^94^. The assay is based on the principle that, when exposed to an electric field, fragmented or damaged DNA migrates more rapidly through a gel matrix than intact DNA^107^. This is because strand breaks reduce the size and cohesion of DNA, allowing smaller fragments to move faster and farther forming a comet-like shape as they migrate from the nucleus. The degree of DNA fragmentation is then measured by analyzing the extent of the comet tail. Interestingly, the alkaline variant of the Comet assay allows the detection of both single- and double-strand damage^108^, while analysis performed in a neutral buffer only enables the detection of double-strand ruptures in DNA^109^. The overestimation of DNA damage due to residual RNA-mediated background ‘noise’, the inability to differentiate endogenous or induced DNA breaks, the lack of standardized protocols and thus the limited data reproducibility between laboratories are listed among the disadvantages of the assay^108,110^. These limitations deem the Comet assay not appropriate for routine control of sperm DNA integrity in animal facilities and sperm laboratories.

The choice of a DNA integrity assay should depend on the characteristics of the mouse strain and the purpose of the spermatological examination, for example, for the routine quality control of sperm before and after cryopreservation, or for the assessment of sperm produced by mice with experimentally induced chromatin disorders. Mouse mutations can affect different phases of sperm chromatin remodeling^111^. Recently, Agudo-Rios et al. employed a wide array of staining methods to compare the DNA integrity, chromatin and protamination status in sperm of three murine species^112^. The investigators highlighted that different techniques might deliver comparable results but are based on different principles and target different aspects of sperm chromatin maturation, compaction and stability^112^. As described above, the diversity of methods used to assess sperm DNA integrity lies not only in their analytical procedure but also in their ability to determine the extent of single-stranded versus double-stranded DNA breaks^95,113^. Given the relationship between the morphokinetics of early embryos and their varying ability to repair single- or double-stranded breaks, it becomes clear that the type of DNA damage is critical for the reproductive outcome^114^. Therefore, it is helpful to consider the potential of an assay to distinguish single- versus double-stranded DNA breaks when selecting a method to assess sperm chromatin status. However, we expect that the choice of such a method would be relevant only to evaluate the fertility of sperm in mouse models with experimentally induced chromatin disorders and not for routine sperm quality control in an animal facility.

## Plasma membrane integrity

The maintenance of the structural and functional integrity of the plasma membrane is a critical milestone for successful sperm cryopreservation as membrane damage during freezing and thawing can cause loss of function or cell death. Specifically, thermal and osmotic shock during cryopreservation can destabilize the plasma membrane and impair a sperm cell’s ability to move, undergo the acrosome reaction and interact with the oocyte, thereby adversely affecting its fertilizing capacity^81^. The susceptibility of sperm plasma membrane to cryoinjury was found to be strain-dependent in mice^6,115^. Furthermore, the capacity of different cryoprotectant agents to improve sperm survival after cryopreservation also varies between mouse strains^81^.

Various stain-based and stain-free methods have been described for assessing sperm plasma membrane integrity, often referred to as ‘sperm viability’ in the literature and in practice. Staining methods can involve either fluorescent or nonfluorescent dyes^116,117^. Based on their binding site and the permeability of the plasma membrane, dyes are grouped as dead-cell markers, that is, dyes that enter the spermatozoon through a damaged membrane, or live-cell markers, that is, dyes that pass through intact membranes and build up in the cytoplasm of the living cells. Moreover, staining tests vary in terms of the required equipment. For example, light or fluorescence microscopes, automated cell counters and flow cytometers are often used for routine evaluation of sperm viability in assisted reproduction laboratories. Researchers in animal facilities can quickly and easily assess sperm viability using nonfluorescent staining combined with phase-contrast microscopy as this approach requires only basic laboratory equipment and minimal training.

The hypo-osmotic swelling test is the most widely used stain-free technique for the assessment of plasma membrane’s functional status and can be performed using a phase-contrast microscope. It is based on the principle that living sperm with functional membranes will swell when exposed to a hypo-osmotic environment due to the influx of fluids^118,119^, with the plasma membrane of the sperm tail exhibiting the most pronounced response to hypo-osmotic stress^119,120^. By calculating the viable/dead cell ratio, this assay can be used to determine viability even in samples with low sperm counts that are usually dedicated for ICSI. However, entirely disrupted sperm cells are not included in the viable/dead cell ratio, which limits the accuracy of the method^121^.

Among the stain-based viability assays, differential staining with eosin/nigrosine^122^ or Trypan blue/Giemsa^122,123^ is considered a simple and cost-efficient technique using brightfield microscopy. Eosin/nigrosin staining reveals the membrane integrity of the sperm head, while Trypan blue/Giemsa staining informs about both the acrosome and the plasma membrane^124^. Furthermore, automated sperm cell counters, such as the NucleoCounter, are devices equipped with an integrated fluorescence microscopy system that can be used not only for the rapid measurement of sperm concentration in a sample but also for the simultaneous evaluation of sperm viability using the fluorescent dye propidium iodide^125^.

Nowadays, flow cytometry is the method of choice for the assessment of sperm viability in many assisted reproduction laboratories. Besides its high repeatability, flow cytometric sperm analysis facilitates the compilation of multicolor fluorescent panels to simultaneously evaluate plasma membrane integrity and other fertility-relevant sperm features, such as acrosomal integrity and mitochondrial function. The list of sperm viability indicators for flow cytometric applications is long and includes Hoechst 33258 (ref. ^126^), YoPro-1 (ref. ^127^), ethidium homodimer-1 (ref. ^128^), ToPro-3 and TOTO^129^, calcein violet^16^ and 7-amino-actinomycin D^130^. The combination of SYBR-14 and propidium iodide is among the most widely used stainings^131,132^. Both dyes bind with DNA to discriminate cells from debris. SYBR-14 is a membrane-permeable dye that stains cells with intact and damaged membranes, while propidium iodide dyes cells with damaged plasma membranes^133^.

## Mitochondrial membrane potential

The functional status of mitochondria, which is critical for energy production and the motility of sperm cells, is closely linked to sperm fertility. Poor mitochondrial function results in low motility and decreased fertilizing capacity^134^. Instead, sperm with high mitochondrial membrane potential (MMP)—the most assessed mitochondrial parameter—have better chances of achieving fertilization of the egg^135^. Mukai et al. showed that mouse spermatozoa remain alive and motile after exposure to chemical drugs blocking oxidative phosphorylation^136^. Therefore, mouse spermatozoa predominantly rely on glycolysis, an anaerobic pathway that does not require oxygen, rather than on oxidative phosphorylation for ATP production^137^. However, a transient increase in oxygen consumption is observed during capacitation, indicating that oxidative phosphorylation in mitochondria becomes more active; therefore, the presence of functional mitochondria is important for the progress and completion of mouse sperm capacitation^138–140^. In addition, MMP also serves as a cryotolerance marker since the impairment of mitochondrial function is one of the first signs of sperm cryoinjury^141^.

The MMP is assessed using potentiometric dyes, which diffuse across the plasma membrane and the outer mitochondrial membrane and accumulate in the inner mitochondrial membrane. An indicative list of the fluorescent dyes used for the evaluation of MMP includes Rhodamine 123, Mitotracker, 5,5′,6,6′-tetrachloro-1,1′,3,3′-tetraethylbenzimidazolyl-carbocyanine iodide (JC-1) and membrane potential-sensitive cyanine dyes, for example, DiIC_1_(5)^16,142,143^. JC-1 shows considerable specificity to mitochondrial membranes and allows the identification of three sperm subpopulations with high, moderate and low MMP^134^. On the other hand, DiIC_1_(5) is convenient for multicolor flow cytometric panels because it is a red laser-excited dye that does not occupy a channel of the blue laser, which is commonly used for the excitation of viability stains or calcium indicators. Fluorescence microscopy or flow cytometry equipment and expertise must be accessible to perform mitochondrial fluorescent staining.

## Capacitation

Capacitation is the second part of post-testicular mammalian sperm maturation, with the first part of the maturation taking place in the epididymis. Capacitation, either induced in the female genital tract or in vitro, gives sperm their ability to approach, bind to and ultimately fuse with the oocyte. It covers major changes in sperm physiology: the biochemical changes allowing an asymmetric pattern of motility, which is characteristic of sperm hyperactivation and necessary for approaching and penetrating the cumulus and zona pellucida; the destabilization of the sperm head plasma membrane and the release of hyaluronidases to facilitate the penetration of the cumulus cells’ barrier; and following acrosome reaction, the partial digestion of the zona pellucida and the fusion of the equatorial segment of sperm plasma membrane with the oocyte membrane^144–149^. Therefore, the initiation, smooth progress and successful completion of capacitation are a prerequisite for both in vivo and in vitro fertilization^149^, with ICSI being an apparent exception in mouse ART^150,151^ as a single spermatozoon is injected directly into a fertile oocyte and does not need to penetrate the cumulus barrier and the zona pellucida. Remarkably, frozen-thawed sperm display premature changes in their ultrastructure and homeostasis similar to the ones occurring during capacitation (‘capacitation-like’ changes, also known as cryocapacitation)^152^. Therefore, assessing the capacitation status of mouse sperm post-thaw also serves as a sign of their freezability.

Given that capacitation comprises a series of events and cannot be assessed as a single functional trait or measurable variable, several ‘proxies’ are usually evaluated for this purpose. These include the percentage of sperm with hyperactivated motility, the reorganization of the lipids in the plasma membrane (for example, the exteriorization of phospholipidic residues such as phosphatidylserine and phosphatidylethanolamine) and the biphasic rise of the cytosolic Ca^2+^ content^153^. In addition, elevated glucose uptake and oxygen consumption rate have been suggested as indicators of (upcoming) capacitation^154^. In this frame, CASA seems to have a decisive advantage compared to subjective motility evaluation with light microscopy as it can provide a detailed insight into the kinematic changes that characterize the hyperactivated motility pattern of capacitated mouse sperm (acquisition of highly vigorous swimming accompanied by deviations from the net direction of movement with turns of ≥90° along the majority of the track)^155,156^.

Regarding plasma membrane structural changes, Merocyanine 540—an indicator of membranal lipid disorders excited by blue light—is the most well-known fluorescent probe targeting phospholipid scrambling and can be combined with several viability stains^127^. The violet ratiometric membrane asymmetry probe also shows promising potential for evaluating capacitation-related plasma membrane destabilization^157,158^. Furthermore, the exteriorization of phosphatidylserine residues during capacitation can be assessed using the fluorochrome-conjugated Annexin-V^159^. However, the interpretation of Annexin-V staining results needs to be done carefully because surface phosphatidylserine exposure can also take place in the course of apoptotic or necrotic processes not related to capacitation^160–162^. The chlortetracycline fluorescence assay, a fluorescence microscopy-based technique, is commonly used to simultaneously evaluate the capacitation status and the acrosome reaction of sperm^163^. The antibiotic chlortetracycline forms strong fluorescent complexes with the Ca^2+^ and Mg^2+^ ions of the membranes of sperm undergoing capacitation^129^; a flow cytometric adaptation of the assay has been also reported^164^. Notably, both Annexin-V and chlortetracycline staining are dependent on Ca^2+^ presence and concentration; thus the composition of the media used for sperm analysis should be carefully checked before these assays^129,160^.

Capacitation is orchestrated through complex signaling pathways, in which several GEM strains can be deficient. These engineered mouse strains exhibit a compromised capacitation status with impaired hyperactivation, decreased mitochondrial respiration and Ca^2+^ content, reduced inducibility of the acrosome reaction or loss of plasma membrane integrity^165,166^. Such strain-related particularities need to be considered when selecting the aspect of capacitation that should be assessed and the appropriate method for this assessment. Furthermore, in contrast to other species in which ejaculated sperm (that is, sperm fully able to capacitate) are used as input for ART, in laboratory mice, it is common to work with epididymal sperm. Caudal sperm already has the ability to capacitate, but this is not the case for caput epididymal sperm, which, therefore, needs to be subjected to an additional in vitro maturation step^167,168^.

The events that should be considered as stages of capacitation, and thus included in capacitation monitoring, have been debated among researchers. For example, Zaneveld et al. considered the reorganization of the membranal phospholipid bilayer as a precursor of acrosome reaction rather than capacitation^169^. Chang argued against the fragmentation of the capacitation phenomena. He suggested including all physiological changes up to the fusion of the male and female gametes under the term ‘capacitation’^170^. In his view, the acrosome reaction should be considered the final stage of capacitation. At the same time, preparatory events leading to hyperactivation and acrosomal exocytosis should be referred to as the first or early stages of capacitation. Gervasi and Visconti put Chang’s view in a molecular context, highlighting the molecular basis of capacitation as an entity that cannot be broken down into distinct phases^171^. However, in both works, it is made clear that researchers and practitioners should refer to the actual phenotype or functional trait they target through the implemented assays and not use the vague categorization of ‘capacitated’ and ‘noncapacitated’ sperm.

## Acrosomal status

The acrosome is a specialized sac-like organelle surrounded by the inner and outer acrosomal membrane and located at the tip of the sperm head. The acrosome contains hydrolytic enzymes (proteinases, phosphatases, acid glycohydrolases, esterases and aryl sulfatases) that are exocytosed before binding with the zona pellucida^172^. Even if a spermatozoon is viable and motile, a compromised or damaged acrosome prevents the sperm cell from fertilizing the egg^173^. Owing to its lability, the acrosome is highly susceptible to damage caused by sperm processing, for example, sex-sorting, storage, centrifugation and cryopreservation.

The sickle-shaped acrosomal cap of mouse sperm can be assessed with simple staining techniques that require only a brightfield microscope, such as staining with Coomassie Blue^174^ or Giemsa^117^. Such staining protocols can be adjusted to simultaneously accommodate a viability stain. This approach enables the quick quantification of viable sperm cells with intact acrosome in a simple laboratory setting. For stain-free evaluation of the acrosomal status, differential interference contrast microscopy must be employed^174^. In laboratories specialized in sperm analysis, fluorescence microscopy and flow cytometry are increasingly used for the determination of acrosomal status^175^. As acrosomes are acidic cellular compartments, the lysosomal stain Lysotracker Green can be used to assess their integrity^176^. However, plant lectins are the most widely used analyte reporters for the acrosome^157,177^. Conjugated with a fluorescent reporter, for example, fluorescein isothiocyanate (FITC), R-phycoerythrin or Alexa Fluor, lectins bind to the carbohydrate moieties of the damaged outer acrosomal membranes. Notably, the *Arachis hypogaea* (peanut) agglutinin, which binds to the β-galactose moieties of the outer acrosomal membrane, is preferred because of its specificity. By contrast, the *Pisum sativum* (edible pea) agglutinin binds to the α-mannose and α-galactose moieties of the acrosomal matrix, head and tail^129^. Combining peanut agglutinin or edible pea agglutinin with a viability indicator enables the detection of sperm populations with intact plasma but compromised acrosome membrane^178,179^. Although less commonly used, Coomassie blue staining has also been reported to be effective for the brightfield microscopic assessment of the acrosome in sperm of humans, bulls, guinea pigs, rabbits, mice, dogs and stallions^174,180^. The status of the acrosome can also be assessed using acrosome-specific antibodies, for example, immunolabeled antibodies for the inner acrosomal membrane protein CD46 (refs. ^181,182^). Nevertheless, as such techniques are laborious and require access to a flow cytometer, they are usually performed for research purposes only and not for routine sperm quality control.

## Limitations and future perspectives

The purpose of sperm quality assessment is to predict fertility by quantifying specific sperm functional characteristics. In the case of cryopreserved sperm, this assessment can also determine if cells have survived the freezing–thawing process and are suitable for IVF. Ideally, well-established thresholds for fertility-relevant sperm characteristics could facilitate reaching this decision. Samples that fail to meet predefined cutoff levels should be considered of lesser fertilizing potential or infertile. It is worth highlighting that, to our knowledge, there are currently no widely accepted quality criteria implemented for cryopreserved mouse sperm. We consider this a critical issue that should be prioritized by the scientific community in the field of mouse-assisted reproduction.

Laboratory mice are bred in a wide variety of strains with diverse genetic backgrounds. Moreover, knockout mouse models with specific anomalies in spermatozoa structure might display compromised sperm quality and reproductive performance. Indeed, features such as sperm motility, morphology and viability considerably vary among different mouse lines^111,183^. Therefore, in some inbred or GEM strains, values of sperm functional traits^81^ or fertilization rates^6^ are suboptimal. In cases of impaired sperm quality post-thaw, one could consider increasing the sperm dose in IVF, implementing sperm motility selection techniques with microfluidics chip sorters^184^ or gel filtration media^80^ and applying zona drilling^185^ as alternatives to improve IVF efficiency. The first alternative is a suitable option for samples with ‘compensable’ sperm defects, when spermatozoa cannot reach or penetrate the oocyte but otherwise carry intact genetic material. The selection of the motile fraction of a sperm population before cryopreservation can considerably improve the functional status and fertility of sperm cells post-thaw^80^. Zona drilling can be applied when dealing with particularly challenging inbred strains that show low IVF efficiency^185^, whereas ICSI remains the method of choice for samples with severely low sperm counts or motility. Nevertheless, high sperm quality does not necessarily guarantee good male fertility; here the limiting factors of the female become critical, such as the quality of the oocyte, the uterine environment and endometrial receptivity, as well as inflammatory conditions, metabolic or hormonal imbalances present in the female animal. Overall, the genetic background and phenotype of a mouse line must be considered when selecting the set of assays performed to evaluate the survival rate and fertility of sperm after cryopreservation. For example, the low sample volume or cell count in oligospermic mice renders full sperm functional analysis infeasible. In such cases, ICSI may be the method of choice for in vitro embryo production; for this method, assessing DNA integrity rather than the motility of sperm is more relevant. On the other hand, assays that target the function of specific sperm organelles are particularly useful in mice carrying mutations that are associated with or affect the status of these organelles. For instance, the assessment of the oxygen consumption rate and mitochondrial activity, both susceptible to cryopreservation stress, can reveal additional information about the cryoability of sperm in mice with modified cation channel function^166^ or experimentally induced metabolic disorders^186^.

In addition to phenotypic indicators of sperm structural and functional integrity, molecular or genetic markers can be used for mouse sperm analysis^85^. However, choosing markers with high predictive potential among the numerous possible markers and genes can be challenging^187,188^. It is worth focusing on these genes that are associated with the cellular response to the shock caused by cryopreservation. Cryoinjury has been linked to alterations in the profile of several sperm biomolecules. Heat shock proteins (HSPs) have been shown to protect against rapid temperature changes and are induced by physical stress. Members of several HSP families are expressed in the testicular tissue and spermatozoa of various species, including mice^189,190^. Notably, elevated levels of *HSP70* (ref. ^191^) and *HSP90* (ref. ^192^) expression have been observed in bovine spermatozoa with heightened cryoresistance and superior post-thaw motion characteristics. Therefore, expression level analysis of these genes may be used for cryotolerance prediction^179,193^; however, relevant studies in mice are still scarce.

Furthermore, sperm with compromised progressive motility after freezing–thawing show decreased levels of both protamine 1 and 2-encoding transcripts in comparison with fresh samples^194^. Genes related to the folding of cytoskeletal proteins can also be involved in the response to cryopreservation-induced stress^195^. Interestingly, to minimize sperm cryoinjury in various animal species, the focus has been shifted to sperm transcriptomics and its link to sperm resilience against cryopreservation stress^196^. Nevertheless, molecular biomarkers of mouse sperm fertility and cryoresistance are still far from being routinely applicable because insight into the functional role of most of these molecules is still incomplete.

Whether opting for functional screening or molecular profiling of sperm, the volume of input data for analysis can be overwhelming. Machine learning tools can be employed to predict the fertilizing potential of spermatozoa by leveraging patterns and relationships in large datasets of measurable attributes associated with sperm quality^197,198^. These algorithms can analyze various sperm parameters and correlate them with fertility data (for example, fertilization rate, blastocyst formation rate and live birth rate) to develop predictive models. A machine learning-based approach should ideally retain only a few markers with a high predictive value altogether. Ultimately, integrated data analysis should aim to facilitate decision-making in laboratory animal facilities and support decisions regarding the suitability of sperm for cryopreservation, the discontinuation of a mouse line or the method of choice for in vitro embryo production. However, certain limitations need to be adequately addressed before implementing machine learning in mouse assisted reproduction and, particularly, sperm fertility prognostics^199^. Namely, the continuous collection and updating of large valid datasets that can be used for algorithm training, while avoiding biases in sperm sample collection, are critical for the development of reliable fertility prognostic algorithms^199^.

## Conclusion

Ensuring the revivability of a mouse line through cryopreserved gametes is critical for deciding when to discontinue animal breeding in a facility. Traditional sperm examination methods often focus on single sperm traits, failing to capture the complexity of sperm function and often providing an incomplete or even misleading picture of sperm fertilizing potential. By contrast, methods such as multicolor flow cytometry and CASA enable the simultaneous analysis of multiple sperm functional and morphometric characteristics, aligning with the multifactorial nature of reproductive success. Integrating multiple traits in sperm quality control provides a more detailed insight into the ability of sperm to maintain their functional status after cryopreservation^200^ and it improves the predictability of the fertility outcome^16,22^. This approach also reflects the true physiological dynamics of sperm subpopulations^155,201^, where compensatory and synergistic mechanisms operate to ensure fertilization. Beyond fertility prediction, the multiparametric analysis of sperm offers a powerful lens into the molecular and physiological alterations underlying reproductive phenotypes particularly in GEM models, where subtle changes may escape detection through isolated or single-trait measures. Taken together, we consider that moving beyond single-trait analyses to a multiparametric approach of mouse sperm quality is not merely an improvement—it is essential for advancing assisted reproduction and reproductive biology in mice.

Realistically, many animal facilities do not have the resources to establish in-house specialized laboratory units for mouse sperm quality control. As a minimum, researchers and facility personnel handling neat sperm samples should be adequately trained for simple procedures to measure sperm concentration, motility and viability using a light microscope before sperm cryopreservation. In addition, documenting sperm quality before freezing is recommended as it serves a dual purpose: (1) to quantify the relative loss of sperm functionality after thawing and (2) to systematically monitor the freezability of a mouse line and the efficiency of the sperm cryopreservation protocols used. To evaluate the survival rate and thus the fertilizing competence of cryopreserved sperm cells, we suggest a more detailed, multiparametric screening of sperm functional status in a dedicated laboratory as the establishment of such units in each animal facility would not be cost-efficient or even feasible. Such a ‘centralized’ solution for mouse sperm quality control would provide a double benefit: access to advanced sperm testing methods for animal facilities and the continuous accumulation of valuable knowledge and expertise regarding mouse sperm fertility evaluation in spermatology laboratories.
